# Ginkgolide B for Myocardial Ischemia/Reperfusion Injury: A Preclinical Systematic Review and Meta-Analysis

**DOI:** 10.3389/fphys.2019.01292

**Published:** 2019-10-16

**Authors:** Peng-Chong Zhu, Qiang Tong, Zhuang Zhuang, Zi-Hao Wang, Li-Hui Deng, Guo-qing Zheng, Yan Wang

**Affiliations:** ^1^Department of Cardiology, The Second Affiliated Hospital and Yuying Children's Hospital of Wenzhou Medical University, Wenzhou, China; ^2^Department of Neurology, The Second Affiliated Hospital and Yuying Children's Hospital of Wenzhou Medical University, Wenzhou, China

**Keywords:** Ginkgolide B, myocardial ischemia/reperfusion injury, possible mechanisms, preclinical evidence, meta-analysis

## Abstract

Ginkgolide B (GB) is an extract of dried Ginkgo biloba leaves and possesses various pharmacological activities in the cardiovascular system. Herein, we aim to assess the available preclinical evidence and possible mechanisms of GB for myocardial ischemia/reperfusion injury. The study quality score was assessed using the CAMARADES 10-item checklist. Rev-Man 5.3 software was used for data analyses. Nineteen studies with total 437 animals were included for analysis. Meta-analyses indicated that GB interventions significantly reduce myocardial infarct size and cardiac markers when compared with control (*P* < *0.05*). The possible mechanisms via which GB exerts cardioprotective effects are mainly associated with anti-oxidation, anti-inflammation, anti-apoptosis, and improvement of energy metabolism. Our study indicates that GB might be a promising cardioprotective agent for myocardial ischemia/reperfusion injury and may contribute to future clinical trial design.

## Introduction

Coronary heart disease (CHD) is the leading cause of death and disability globally (Ibanez et al., 2015). Acute myocardial infarction (AMI) is one critical clinical presentation of coronary artery disease (Yellon and Hausenloy, 2007). Early restoration of blood flow through thrombolysis or primary percutaneous coronary intervention has been proved to be the most effective method to rescue ischemic myocardium, reduce infarct size, and improve the clinical outcomes (Frank et al., 2012). However, the process of reperfusion paradoxically lead to myocardial ischemia/reperfusion (I/R) injury such as myocardial necrosis, stunning, heart failure, no-reflow and reperfusion arrhythmias (Binder et al., 2015), which would undermine the benefits of myocardial reperfusion (Minamino, 2012). Myocardial I/R injury is an intricate phenomenon involved in many factors, all conducing to the final damage inflicted on the cardiomyocyte (Bulluck et al., 2016). Over the past 3 decades, numerous therapeutic strategies, both pharmacologic and non-pharmacologic, have been proposed to ameliorate the myocardial I/R injury in animal experiments. However, the basic research yielded inconclusive results or failed to translate into the clinical setting efficiently (Eltzschig and Eckle, 2011). Hence, it is worthy to find a novel cardioprotective therapeutic intervention to prevent further tissue injury caused by reperfusion.

**Figure 1:**
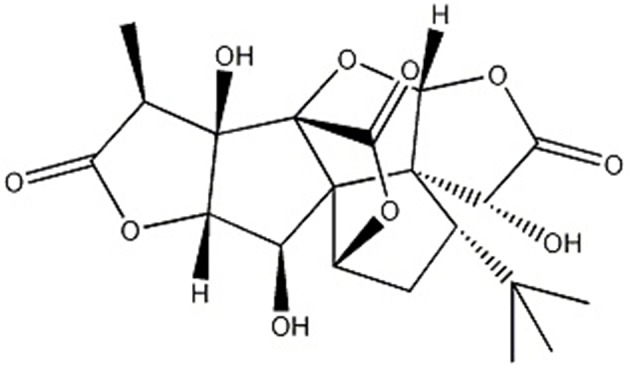
Chemical structures of GB (C_20_H_24_O_10_).

*Ginkgo biloba* L., known as “living fossil” in plant kingdom (Jacobs and Browner, 2000), its leaves were applied as a traditional Chinese medicine (TCM) for the management of cardiovascular disorders for hundreds of years because of its properties of promoting blood circulation to remove blood stasis (Zhang et al., 2016) and activating meridians to stop pain (Li et al., 2017). Ginkgolide B (GB) (Figure 1), an extract of dried Ginkgo biloba leaves, was discovered as a natural antagonist of platelet activating factor (PAF) with various biological and pharmacological functions, including anti-allergic (Bilia, 2002), anti-inflammation (Xia and Fang, 2007), and anti-oxidation (Maclennan et al., 2002). Thus, it was widely used for I/R diseases including AMI.

The animal research has its special primary purpose in improving the understanding of physiologic and pathologic processes (Briel et al., 2013). Systematic reviews of preclinical studies play an important role in healthcare practice which can help promote the translation of findings from animal studies to clinical trials rapidly and appropriately, and avoid unintended waste of time (Sandercock and Roberts, 2002). In addition, preclinical systematic review can contribute to illustrate mechanisms, synthesize the available evidence, support clinical decision-making, and improve the methodological quality of experiments and guide health policy (Roberts et al., 2002). Many animal studies have hitherto investigated the role of GB in myocardial I/R injury. However, the efficacy and mechanisms of GB for myocardial I/R injury have not been systematically appraised and summarized. In the present study, we performed a systematic review for these animal studies to assess the available preclinical evidence and possible mechanisms of GB in treating myocardial I/R injury.

## Methods

### Data Sources and Search Strategy

The PRISMA checklist is available as supporting information (Stewart et al., 2015). We searched for animal studies of GB for myocardial I/R injury in Chinese National Knowledge Infrastructure (CNKI), EMBASE, PubMed, VIP information database, Web of Science, and Wanfang data Information Site published up to the end of April 2019. The following search phrases were used: “Yinxingneizhi B (MeSH Terms) OR Ginkgolide B (Title/Abstract)” AND “Myocardial infarction OR Myocardial ischemia OR Myocardial I/R”. The references of the included articles cited were searched manually for the eligible studies.

### Eligibility Criteria

Studies were included for analysis when they meet: (1) animal studies assessing the administration of GB for myocardial I/R model *in vivo* or *ex vivo* except cell studies, case reports, reviews, abstracts, and comments; (2) analyzed intervention received GB treatment merely; comparator intervention received vehicle or no treatment; (3) primary outcomes were myocardial infarction (MI) size, myocardial injury marker, ST-segment changes in electrocardiograms, or cardiac function indicators in cardiac ultrasound, while secondary outcomes were serum indices or protein levels relative to the mechanisms of GB in myocardial I/R injury. The studies compared with other TCM, administered other additional pharmacological treatment, without predetermined outcome index and without involving animal models of myocardial I/R injury were excluded.

### Data Extraction

The following details were recorded: (1) first author's name and year of publication; (2) animal information, including species, gender, number, and weight; (3) methods for myocardial I/R model establishment, including I/R duration and coronary artery occluded; (4) method for anaesthetic induction; (5) the information of treatment, including time of intervention, dose, and route of administration; (6) the primary outcome measures, secondary outcome measures and its intergroup differences. Only the data of final test was collected when outcome measures were tested at different time points. The result of highest dose was extracted if various doses of drugs were used in the study. If some data were only expressed as graphs, we reached out to the authors for raw data. Measure numerical values by digital ruler software when no response was got from authors.

### Risk of Bias in Individual Studies

Two authors independently evaluated the quality of study using the 10-point scoring scale (Macleod et al., 2004): A, peer-reviewed publication; B, statement of temperature control; C, random allocation to groups; D, allocation concealment; E, blinded assessment of outcome; F, use of anesthetic without significant intrinsic cardioprotective activity; G, appropriate animal model (aged, diabetic, or hypertensive); H, sample size calculation; I, compliance with animal welfare regulations; and J, statement of potential conflict of interests. Study selection, data extraction, and quality evaluation of studies were independently performed by two authors, and the disputes were discussed in group discussion.

### Statistical Analysis

All outcome measures were treated as continuous data. Every outcome with at least 2 available studies was analyzed with RevMan V.5.3 software. Outcomes were presented as Standardized mean difference (SMD) with 95% confidence interval (CI). Heterogeneity among the studies was evaluating using the *I*-square (*I*^2^) statistics test. If *I*^2^> 50%, a random effects model was applied; otherwise, a fixed effects model was applied. *P*-value <0.05 was considered significant. A forest plot was created to summarize the meta-analysis study results.

## Results

**Figure 2:**
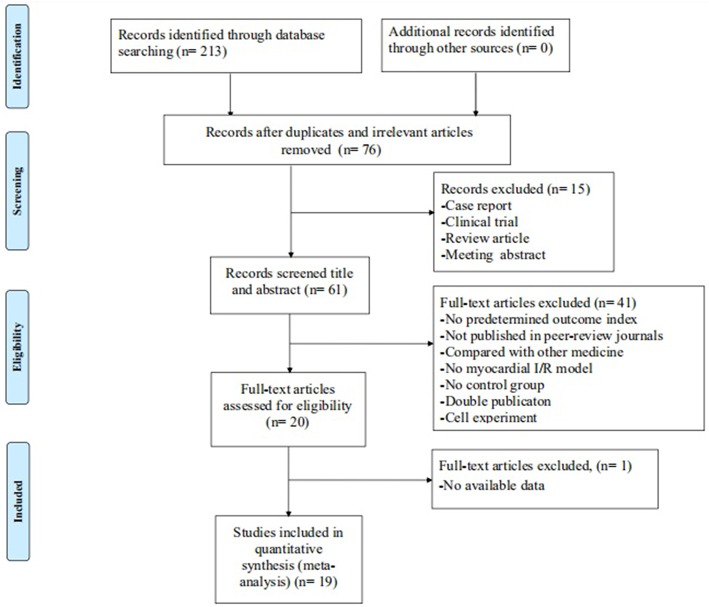
Flow diagram of the systematic review.

The initial search yielded a total of 213 studies. After excluding 137 irrelevant and duplicated studies, the full texts of 76 studies were screened. In the process, 57 studies were excluded according to the inclusion and exclusion criteria. Ultimately, 19 studies (Liebgott et al., 2000; Jiao et al., 2002; Rioufol et al., 2003; Billottet et al., 2005; Zhang et al., 2005, 2018; Hao et al., 2009, 2013, 2016; Zhao et al., 2014; Pei et al., 2015; Xiong and Wei, 2015; Bai et al., 2016; Cao et al., 2016; Hao and Dong, 2017; Meng, 2017; Zhuang et al., 2017; Chai et al., 2018; Yang et al., 2018) were selected. A detailed step of the selection process is shown in Figure 2.

### Characteristics of Included Studies

Twelve Chinese studies (Zhang et al., 2005; Hao et al., 2013, 2016; Zhao et al., 2014; Xiong and Wei, 2015; Bai et al., 2016; Cao et al., 2016; Hao and Dong, 2017; Meng, 2017; Zhuang et al., 2017; Chai et al., 2018; Yang et al., 2018) and 7 English studies (Liebgott et al., 2000; Jiao et al., 2002; Rioufol et al., 2003; Billottet et al., 2005; Hao et al., 2009; Pei et al., 2015; Zhang et al., 2018) were published between 1996 and 2018. Male Sprague-Dawley rats (11 studies) (Hao et al., 2009, 2013, 2016; Xiong and Wei, 2015; Bai et al., 2016; Hao and Dong, 2017; Meng, 2017; Zhuang et al., 2017; Chai et al., 2018; Yang et al., 2018; Zhang et al., 2018), male/female Sprague-Dawley rats (2 studies) (Zhao et al., 2014; Cao et al., 2016), male Wistar rats (4 studies) (Liebgott et al., 2000; Jiao et al., 2002; Billottet et al., 2005; Pei et al., 2015), male/female Wistar rats (1 study) (Zhang et al., 2005), and male/female Farm pigs (1 study) (Rioufol et al., 2003)were utilized. Chloral hydrate was used in 4 studies (Zhao et al., 2014; Xiong and Wei, 2015; Yang et al., 2018; Zhang et al., 2018); pentobarbital sodium in 7 studies (Liebgott et al., 2000; Billottet et al., 2005; Hao et al., 2013, 2016; Pei et al., 2015; Cao et al., 2016; Hao and Dong, 2017); urethane in 6 studies (Jiao et al., 2002; Zhang et al., 2005; Bai et al., 2016; Meng, 2017; Zhuang et al., 2017; Chai et al., 2018); pentobarbitalin 2 studies (Rioufol et al., 2003; Hao et al., 2009) for anesthesia. Ligating the coronary artery was to induce the model of I/R, of which the left anterior descending coronary artery (LAD) was in 11 studies (Jiao et al., 2002; Rioufol et al., 2003; Pei et al., 2015; Xiong and Wei, 2015; Bai et al., 2016; Cao et al., 2016; Meng, 2017; Zhuang et al., 2017; Chai et al., 2018; Yang et al., 2018; Zhang et al., 2018). Others were established by the langendorff method (Liebgott et al., 2000; Billottet et al., 2005; Zhang et al., 2005; Hao et al., 2009, 2013, 2016; Zhao et al., 2014; Hao and Dong, 2017). MI size as outcome was reported in 9 studies (Jiao et al., 2002; Hao et al., 2009, 2016; Xiong and Wei, 2015; Bai et al., 2016; Cao et al., 2016; Meng, 2017; Zhuang et al., 2017; Zhang et al., 2018); ejection fraction (EF) in 2 studies (Xiong and Wei, 2015; Meng, 2017), fractional shorting (FS) in 2 studies (Xiong and Wei, 2015; Meng, 2017); Maximal rate of the increase/decrease of left ventricular pressure (±dp/dtmax) in 8 studies (Jiao et al., 2002; Hao et al., 2009, 2013, 2016; Zhao et al., 2014; Pei et al., 2015; Hao and Dong, 2017; Yang et al., 2018); left ventricular developed pressure (LVDP) in 3 studies (Liebgott et al., 2000; Billottet et al., 2005; Yang et al., 2018); left ventricular end diastolic pressure (LVEDP) in 4 studies (Liebgott et al., 2000; Billottet et al., 2005; Hao et al., 2009, 2016); myocardial cell apoptosis index (AI) in 4 studies (Jiao et al., 2002; Bai et al., 2016; Meng, 2017; Zhuang et al., 2017); arrhythmia score in 3 studies (Zhang et al., 2005; Pei et al., 2015; Chai et al., 2018). Level of lactate dehydrogenase (LDH) was reported in 9 studies (Hao et al., 2009, 2013, 2016; Zhao et al., 2014; Pei et al., 2015; Bai et al., 2016; Cao et al., 2016; Hao and Dong, 2017; Meng, 2017); creatine kinase (CK) in 4 studies (Zhang et al., 2005; Bai et al., 2016; Cao et al., 2016; Meng, 2017) superoxide dismutase (SOD) in 5 studies (Zhang et al., 2005; Hao et al., 2013; Bai et al., 2016; Cao et al., 2016; Chai et al., 2018); malondialdehyde (MDA) in 4 studies (Zhang et al., 2005; Bai et al., 2016; Cao et al., 2016; Chai et al., 2018); glutathione peroxidase (GSH-Px) in 2 studies (Bai et al., 2016; Chai et al., 2018); caspase-3 in 2 studies (Zhuang et al., 2017; Yang et al., 2018); B-cell lymphoma-2 (Bcl-2) in 4 studies (Hao et al., 2009, 2016; Xiong and Wei, 2015; Zhuang et al., 2017) and Bcl-2-Associated X (Bax) in 4 studies (Hao et al., 2009, 2016; Xiong and Wei, 2015; Zhuang et al., 2017); aspartate transaminase (AST) in 2 studies (Bai et al., 2016; Meng, 2017). Nuclear factor-kappaB (NF-kB) was mentioned in 2 study (Bai et al., 2016; Zhang et al., 2018); p38-mitogen-activated protein kinase (p38-MAPK), extracellular signal-regulated kinase (ERK) and c-Jun N-terminal kinase (JNK) in 1 study (Xiong and Wei, 2015); coronary flow (CF) in 1 study (Billottet et al., 2005); segment shortening in 1 study (Rioufol et al., 2003), mean arterial pressure (MAP) in 1 study (Rioufol et al., 2003), and tumor necrosis factor-α (TNF-α) and interleukin-6 (IL-6) in 1 study (Zhang et al., 2018). The characteristics of selected study is provided in Table 1.

**Table 1:** Characteristics of the 19 included studies.

**Study (years)**	**Species (Sex, *n* = experimental/control group)**	**Weight**	**Model (method)**	**Anesthetic**	**Treatment group (method to GB)**	**Control group**	**Outcome index (time)**	**Intergroup differences**
Zhuang et al., 2017 (1)	SD rats (male, 20/20)	220–260 g	Block LAD for 30 min then reperfusion	Urethane (650 mg/kg)	Intraperitoneal injection GB (60 mg/kg*d), once a day, for 7 days, after establishing model	Intraperitoneal injection isasteric normal saline, once a day, for 7 days, after establishing model	Infarct size (AAI/LVA) Apoptosis index Bcl-2 Bax Caspase-3	*P* < 0.01 *P* < 0.01 *P* < 0.01 *P* < 0.01 *P* < 0.01
Xiong and Wei, 2015 (2)	SD rats (male, 20/20)	250–270 g	Block of LAD for 30 min then reperfusion for 48 h	7% chloral hydrate	Gavaged with GB (60 mg/kg*d), once a day, for 7 days, before establishing model	Gavaged with isasteric normal saline, once a day, for 7 days, before establishing model	Infarct size (AAI/LVA) EF FS Bcl-2 Bax p-ErK 7.p-JNK p-p38MAPK	*P* < 0.01 *P* < 0.01 *P* < 0.01 *P* < 0.01 *P* < 0.01 *P* < 0.01 *P* < 0.01 *P* < 0.01
Cao et al., 2016 (3)	SD rats (male/female, 10/10)	200–240 g	Block LAD for 30 min then reperfusion for 2 h	Pentobarbital sodium (45 mg/kg)	Tail intravenous injection GB (8 mg/kg), 1 h before establishing model	Tail intravenous injection isasteric normal saline, 1 h before establishing model	Infarct size (AAI/LVA) CK LDH SOD MDA	*P* < 0.05 *P* < 0.01 *P* < 0.01 *P* < 0.01 *P* < 0.01
Meng, 2017 (4)	SD rats (male, 20/20)	220–260 g	Block LAD for 30 min then reperfusion	Urethane (650 mg/kg)	Intraperitoneal injection GB (60 mg/kg*d), once a day, for 7 days, after establishing model	Intraperitoneal injection isasteric normal saline, once a day, for 7 days, after establishing model	Infarct size (AAI/LVA) EF FS LVIDd LVIDs SV Apoptosis index AST CK LDH	*P* < 0.01 *P* < 0.01 *P* < 0.01 *P* < 0.01 *P* < 0.01 *P* < 0.01 *P* < 0.01 *P* < 0.01 *P* < 0.01
Bai et al., 2016 (5)	SD rats (male, 20/20)	220–260 g	Block LAD for 30 min then reperfusion	Urethane (650 mg/kg)	Intraperitoneal injection GB (60 mg/d), once a day, for 7 days, after establishing model	Intraperitoneal injection isasteric normal saline, once a day, for 7 days, after establishing model	Infarct size (AAI/LVA) Apoptosis index SOD GSH-Px CAT MDA AST CK LDH NF-kB	*P* < 0.01 *P* < 0.01 *P* < 0.01 *P* < 0.01 *P* < 0.01 *P* < 0.01 *P* < 0.01 *P* < 0.01 *P* < 0.01 *P* < 0.01
Chai et al., 2018 (6)	SD rats (male, 20/20)	220–260 g	Block LAD for 30 min then reperfusion for 1 h	Urethane (1 g/kg)	Intraperitoneal injection GB (60 mg/kg*d), once a day, for 7 days, before establishing model	Intraperitoneal injection (18 mg/kg*d) normal saline, once a day, for 7 days, before establishing model	Arrhythmia score SOD GSH-Px MDA	*P* < 0.01 *P* < 0.01 *P* < 0.01 *P* < 0.01
Yang et al., 2018 (7)	SD rats (male, 7/7)	180–220 g	Block LAD for 30 min then reperfusion for 2 h	10% chloral hydrate	Intraperitoneal injected with GB (60 mg/kg), 30 min before establishing model	Intraperitoneal injection isasteric normal saline, 30 min before establishing model	LVSP LVDP +dP/dtmax –dP/dtmax Caspase-3	*P* < 0.05 *P* < 0.05 *P* < 0.05 *P* < 0.05 *P* < 0.05
Jiao et al., 2002 (8)	Wistar rats (male, 12/12)	250–300 g	Block LAD for 30 min then reperfusion for 3 h	Urethane (1 g/kg)	Intraperitoneal injection GB (5 mg/kg), 25 and 10 min before establishing model respectively	Intraperitoneal injection isasteric normal saline, 25 and 10 min before establishing model respectively	Infarct size (AAI/AAR) LVSP +dP/dtmax –dP/dtmax Apoptosis index	*P* < 0.05 *P* > 0.05 *P* < 0.05 *P* > 0.05 *P* < 0.05
Rioufol et al., 2003 (9)	Farm pigs (male/female, 6/7)	27–28 Kg	Block LAD for 10 min then reperfusion for 3 h	Pentobarbital (15 mg/kg)	Intravenous injection GB (0.3 mg/kg), 48 h and 24 h before establishing model	Intravenous injection isasteric normal saline, 48 h and 24 h before establishing model	HR Mean arterial pressure Segment shortening	*P* > 0.05 *P* > 0.05 *P* > 0.05
Pei et al., 2015 (10)	Wistar rats (male, 10/12)	280–400 g	Block LAD for 5 min then reperfusion for 10 min	pentobarbitalsodium (4.5 mg/kg)	Intravenous injection GB (15 mg/kg), 10 min before establishing model	Intravenous injection isasteric normal saline, 10 min before establishing model	LVSP Arrhythmia score +dP/dtmax –dP/dtmax LDH	*P* < 0.01 *P* < 0.01 *P* < 0.01 *P* < 0.01 *P* < 0.05
Hao et al., 2009 (11)	SD rats (male, 7/7)	220–250 g	Langendorff Model 30 min stabilization I/R (30 min/120 min)	Pentobarbital (150 mg/kg)	GB (10uM), for 10 min before ischemia	No treatment	Infarct size (AAI/LVA) HR LVSP LVEDP +dP/dtmax –dP/dtmax LDH Bcl-2 Bax	*P* < 0.05 *P* < 0.01 *P* > 0.05 *P* > 0.05 *P* < 0.05 *P* < 0.05 *P* < 0.05 *P* > 0.05 *P* > 0.05
Hao and Dong, 2017 (12)	SD rats (male, 10/10)	350–500 g	Langendorff Model 30 min, I/R (30 min/60 min)	1% pentobarbital sodium	GB (10 μmol/L), first 10 min of reperfusion	No treatment	ΔLVP (LVSP -LVEDP) +dP/dtmax –dP/dtmax LDH	*P* < 0.05 *P* < 0.05 *P* < 0.05 *P* < 0.05
Hao et al., 2013 (13)	SD rats (male, 8/8)	200–250 g	Langendorff Model 30 min stabilization I/R (20 min/40 min)	1% pentobarbital sodium	GB (10 μmol/L), for 10 min before ischemia	No treatment	ΔLVP (LVSP -LVEDP) +dP/dtmax –dP/dtmax HR LDH SOD	*P* < 0.01 *P* < 0.05 *P* < 0.01 *P* < 0.05 *P* < 0.01 *P* < 0.05
Hao et al., 2016 (14)	SD rats (male, 10/10)	350–500 g	Langendorff Model 30 min stabilization I/R (30 min/90 min)	1% pentobarbital sodium	GB (10 μmol/L), first 10 min of reperfusion	No treatment	Infarct size (AAI/LVA) LVSP LVEDP +dP/dtmax –dP/dtmax LDH Bcl-2 Bax	*P* < 0.05 *P* > 0.05 *P* < 0.05 *P* < 0.05 *P* < 0.05 *P* < 0.01 *P* < 0.05 *P* < 0.05
Liebgott et al., 2000 (15)	Wistar rats (male, 12/13)	300–350 g	Langendorff Model 30 min stabilization, low-flow ischemia/I/R (10 min/30 min/60 min)	Pentobarbital sodium (50 mg/kg)	GB (0.05 μg/ml), last 10 min of perfusion, entire period of low-flow ischemia and the first 10 min of reperfusion	No treatment	LVDP LVEDP RPP LVdP/dt CF	*P* < 0.02 *P* < 0.02 *P* < 0.02 *P* < 0.02 *P* < 0.02
Zhao et al., 2014 (16)	SD rats (male/female, 10/10)	200–250 g	Langendorff Model 30 min stabilization I/R (20 min/40 min)	10% chloral hydrate	GB (10 μmol/L), for 10 min before ischemia	No treatment	ΔLVP (LVSP -LVEDP) +dP/dtmax –dP/dtmax LDH	*P* < 0.01 *P* < 0.05 *P* < 0.01 *P* < 0.01
Zhang et al., 2005 (17)	Wistar rats (male/female, 8/8)	200–250 g	Langendorff Model 30 min stabilization, I/R (15 min/30 min)	20% Urethane (1.0 g/kg)	GB (10 μmol/L), for 10 min before ischemia	No treatment	Arrhythmia score LDH CK SOD MDA	*P* < 0.01 *P* < 0.05 *P* < 0.05 *P* < 0.05 *P* < 0.01
Billottet et al., 2005 (18)	Wistar rats (male, 12/12)	300–350 g	Langendorff Model 30 min stabilization, low-flow ischemia/I/R (20 min/20 min/60 min)	Pentobarbital sodium (50 mg/kg)	GB (0.05 μg/ml), last 10 min of perfusion, entire period of low-flow ischemia and the first 10 min of reperfusion	No treatment	LVDP LVEDP RPP LVdP/dt CF	*P* < 0.05 *P* < 0.05 *P* < 0.05 *P* < 0.05 *P* < 0.05
Zhang et al., 2018 (19)	SD rats (male, 8/8)	250–300 g	Block LAD for 40 min then reperfusion for 2 h	Chloral hydrate (300 mg/kg)	Intraperitoneal injection GB (32 mg/kg*d), once a day, for 7 days, before establishing model	Intraperitoneal injection isasteric normal saline, once a day, for 7 days, after establishing model	Infarct size (AAI/AAR) histopathological scores PMNs TNF-α, IL-6 MPO	*P* < 0.05 *P* < 0.05 *P* < 0.05 *P* < 0.05 *P* < 0.05 *P* < 0.05

*AAI, Area at infarct; AAR, area at risk; AST, aspartate transaminase; Bax, Bcl-2-Associated X; Bcl-2, B-cell lymphoma-2; CAT, catalase; CK, creatine kinase; EF, ejection fraction; ErK, extracellular signal-regulated kinase; FS, fractional shorting; GSH-Px, glutathione peroxidase; HR, heart rate; IL-6, interleukin-6; JNK, c-Jun N-terminal kinase; LAD, left anterior descending coronary artery; LVA, left ventricular area; LVDP, left ventricular developed pressure; LVEDP, left ventricular end diastolic pressure; LDH, lactate dehydrogenase; LVIDd, left ventricular internal diameter at end-diastole; LVIDs, left ventricular internal diameter at end-systole; LVSP, Left ventricular systolic pressure; MDA, malondialdehyde; MPO, myeloperoxidase; ±dp/dtmax, Maximal rate of the increase/decrease of left ventricular pressure; p38MAPK, p38 mitogen-activated protein kinase; NF-kB, Nuclear factor-kappa B; PMNs, polymorphonuclears; RPP, rate pressure pruduct; SOD, superoxide dismutase; SV, stroke volume; SD rats, Sprague-Dawley; TNF-α, tumor necrosis factor-α*.

### Study Quality

**Figure 3:**
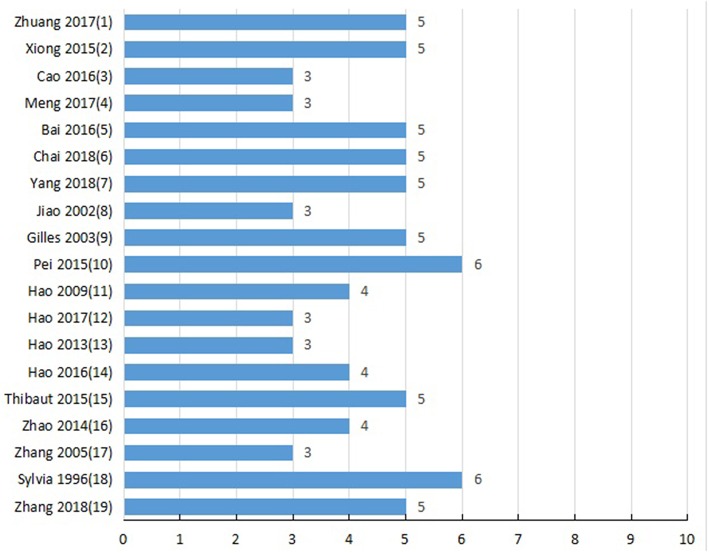
Methodological quality for each study included.

Detailed results of methodological quality are presented in Table 2 and Figure 3. All studies were peer-reviewed. Use of anesthetic without significant intrinsic cardioprotective activity were all described. Eleven studies (Liebgott et al., 2000; Rioufol et al., 2003; Billottet et al., 2005; Hao et al., 2009; Pei et al., 2015; Xiong and Wei, 2015; Bai et al., 2016; Zhuang et al., 2017; Chai et al., 2018; Yang et al., 2018; Zhang et al., 2018) reported control of temperature. None of the studies discussed allocation concealment, blinded assessment of outcome and the sample size calculation. No study choose the appropriate animal model. Compliance with animal welfare regulations was stated in 12 studies (Liebgott et al., 2000; Rioufol et al., 2003; Billottet et al., 2005; Zhao et al., 2014; Pei et al., 2015; Xiong and Wei, 2015; Bai et al., 2016; Hao et al., 2016; Zhuang et al., 2017; Chai et al., 2018; Yang et al., 2018; Zhang et al., 2018) and statement of potential conflict of interests was declared in 2 studies (Pei et al., 2015; Zhang et al., 2018).

**Table 2:** Risk of bias of the included studies.

**Study**	**A**	**B**	**C**	**D**	**E**	**F**	**G**	**H**	**I**	**J**	**Total**
Zhuang et al., 2017 (1)	+	+	+	–	–	+	–	–	+	–	5
Xiong and Wei, 2015 (2)	+	+	+	–	–	+	–	–	+	–	5
Cao et al., 2016 (3)	+	–	+	–	–	+	–	–	–	–	3
Meng, 2017 (4)	+	–	+	–	–	+	–	–	–	–	3
Bai et al., 2016 (5)	+	+	+	–	–	+	–	–	+	–	5
Chai et al., 2018 (6)	+	+	+	–	–	+	–	–	+	–	5
Yang et al., 2018 (7)	+	+	+	–	–	+	–	–	+	–	5
Jiao et al., 2002 (8)	+	–	+	–	–	+	–	–	+	–	3
Rioufol et al., 2003 (9)	+	+	+	–	–	+	–	–	+	–	5
Pei et al., 2015 (10)	+	+	+	–	–	+	–	–	+	+	6
Hao et al., 2009 (11)	+	+	+	–	–	+	–	–	+	–	4
Hao and Dong, 2017 (12)	+	–	+	–	–	+	–	–	+	–	3
Hao et al., 2013 (13)	+	–	+	–	–	+	–	–	+	–	3
Hao et al., 2016 (14)	+	–	+	–	–	+	–	–	+	–	4
Liebgott et al., 2000 (15)	+	+	+	–	–	+	–	–	+	–	5
Zhao et al., 2014 (16)	+	–	+	–	–	+	–	–	+	–	4
Zhang et al., 2005 (17)	+	–	+	–	–	+	–	–	+	–	3
Billottet et al., 2005 (18)	+	+	+	–	–	+	–	–	+	+	6
Zhang et al., 2018 (19)	+	+	+	–	–	+	–	–	+	–	5

*Studies fulfilling the criteria of: A, peer reviewed publication; B, control of temperature; C, random allocation to treatment or control; D, blinded induction of model; E, blinded assessment of outcome; F, use of anesthetic without significant intrinsic cardioprotective activity; G, appropriate animal model (aged, diabetic, or hypertensive); H, sample size calculation; I, compliance with animal welfare regulations; J, statement of potential conflict of interests*.

### Effectiveness

#### Primary Outcome Measures

##### Myocardial infarction size

**Figure 4:**
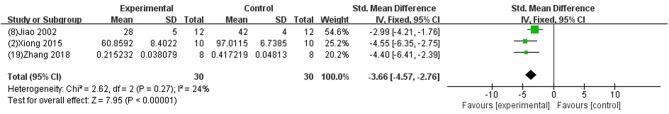
Forest plot of animal studies investigating the effect of GB on myocardial infarction size (AAI/AAR) reduction.

**Figure 5:**
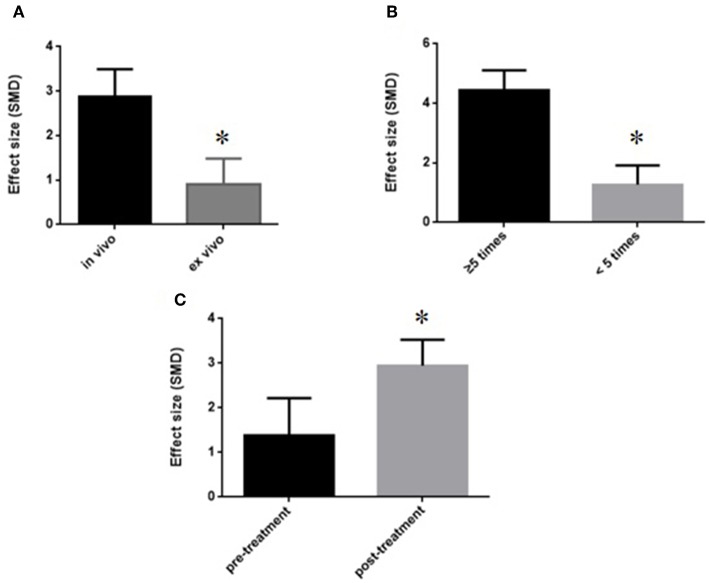
Subgroup analyses of the myocardial infarct size (infarct area/left ventricular area). **(A)** Experimental model; **(B)** stage of GB administration; **(C)** frequency of drug administration. The magnitude of absolute value SMD reflected the effect size. *P < 0.05 vs. control groups.

We divided the studies into two parts by different calculation methods: (1) Area at infarct/area at risk (AAI/AAR): meta-analysis of 3 studies (Jiao et al., 2002; Xiong and Wei, 2015; Zhang et al., 2018) indicated that MI size was significantly reduced using GB compared with control (SMD −3.66, 95% CI [−4.57 to −2.76], *P* < 0.00001; *I*^2^ = 24%) (Figure 4); (2) Area at infarct/left ventricular area (AAI/LVA): meta-analysis of 6 studies (Hao et al., 2009, 2016; Bai et al., 2016; Cao et al., 2016; Meng, 2017; Zhuang et al., 2017) indicated that MI size was significantly reduced by using GB compared with control (SMD −2.52, 95% CI [−3.01 to −2.04], *P* < 0.00001; *I*^2^ = 89%). There was high statistical heterogeneity among comparison of MI size (AAI/LVA). We performed a sensitivity analysis, the result remained after excluding each of these studies. In addition, subgroup analysis is layered with the following groups: experimental model (*in vivo* or *ex vivo*), stage of GB administration (pre-treatment and post-treatment), and frequency of drug administration. GB prevented MI size more obviously at the model *in vivo* than the model *ex vivo* (SMD = −2.91 vs. SMD = −0.98, *P* < 0.05, Figure 5A). Groups with more times (≥5) of administration of GB showed a better outcome than that with fewer times (<5) (SMD = −4.13 vs. SMD = −1.23, *P* < 0.05, Figure 5B). Compared with giving GB after the model, the GB pre-treatment (before introducing the model) did not produce a better outcome (SMD = −1.37 vs. SMD = −3.00, *P* < 0.05, Figure 5C).

##### Cardiac function and the level of ST-segment elevation

**Figure 6:**
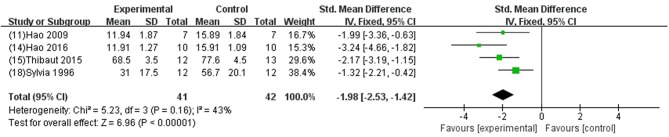
Forest plot of animal studies investigating the effect of GB on LVEDP decrease.

GB was significant for improving EF and FS (*P* < 0.05) (Xiong and Wei, 2015; Meng, 2017); increasing +dp/dtmax and LVSP (Jiao et al., 2002; Hao et al., 2009, 2013, 2016; Zhao et al., 2014; Pei et al., 2015; Hao and Dong, 2017; Yang et al., 2018) in systolic function (*P* < 0.05); increasing –dp/dtmax (Jiao et al., 2002; Hao et al., 2009, 2013, 2016; Zhao et al., 2014; Pei et al., 2015; Hao and Dong, 2017; Yang et al., 2018) and LVDP (Liebgott et al., 2000; Billottet et al., 2005; Yang et al., 2018) (*P* < 0.05), and decreasing LVEDP (SMD−1.98, 95% CI [−2.53 to −1.42], *P* < 0.00001; *I*^2^ = 43%) (Figure 6) in diastolic function; decreasing the arrhythmia score (Zhang et al., 2005; Pei et al., 2015; Chai et al., 2018) (*P* < 0.05); and reducing the ST segment elevation (Chai et al., 2018) (*P* < 0 05), when compared with control.

##### Myocardial injury marker

**Figure 7:**
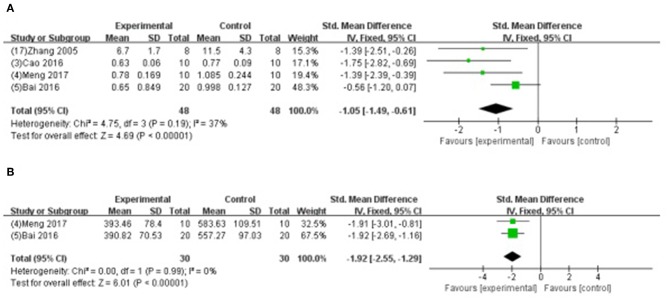
**(A)** Forest plot of animal studies investigating the effect of GB on creatine kinase decrease. **(B)** Forest plot of animal studies investigating the effect of GB on aspartate transaminase decrease.

Meta-analysis of 4 studies (Zhang et al., 2005; Bai et al., 2016; Cao et al., 2016; Meng, 2017) indicated GB was significant for decreasing the level of CK using (SMD −1.05, 95% CI [−1.49 to −0.61], *P* < 0.00001; *I*^2^ = 37%) (Figure 7A); and 2 studies (Bai et al., 2016; Meng, 2017) for decreasing the level of AST (SMD −1.92, 95% CI [−2.55 to −1.29], *P* < 0.00001; *I*^2^ = 0%) (Figure 7B) compared with control. Level of LDH was measured in 9 studies (Hao et al., 2009, 2013, 2016; Zhao et al., 2014; Pei et al., 2015; Bai et al., 2016; Cao et al., 2016; Hao and Dong, 2017; Meng, 2017), and all of them indicated GB was significant for reducing the level of LDH (*P*<*0.05*) compared with control.

#### Secondary Outcome Measures

**Figure 8:**
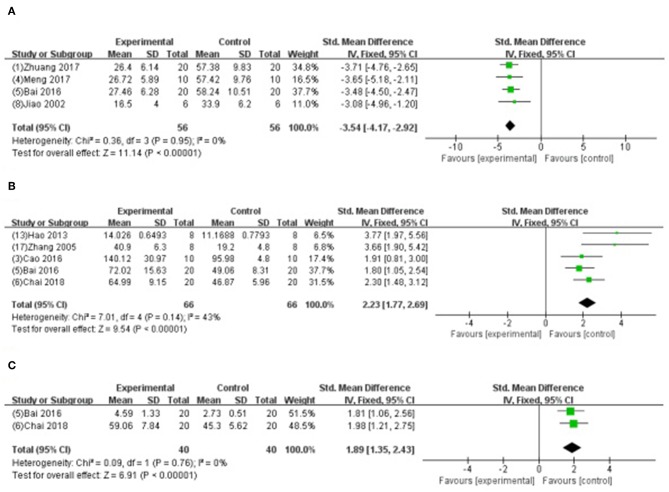
**(A)** Forest plot of animal studies investigating the effect of GB onapoptosis index decrease. **(B)** Forest plot of animal studies investigating the effect of GB onsuperoxide dismutasedecrease. **(C)** Forest plot of animal studies investigating the effect of GB on glutathione peroxidase.

Four studies (Jiao et al., 2002; Bai et al., 2016; Meng, 2017; Zhuang et al., 2017) showed that GB-treated group was superior to the untreated group according to the reduced AI (*n* = 56, SMD −3.54, 95% CI [−4.17 to −2.92], *P* < 0.00001; *I*^2^ = 0%) (Figure 8A); 5 studies (Zhang et al., 2005; Hao et al., 2013; Bai et al., 2016; Cao et al., 2016; Chai et al., 2018) increasing SOD (SMD −2.23, 95% CI [1.77 to 2.69], *P* < 0.00001; *I*^2^ = 43%) (Figure 8B); 2 studies (Bai et al., 2016; Chai et al., 2018) increasing GSH-Px (SMD 1.89, 95% CI [1.35 to 2.43], *P* < 0.00001; *I*^2^ = 0%) (Figure 8C); 4 studies (Zhang et al., 2005; Bai et al., 2016; Cao et al., 2016; Chai et al., 2018) reducing MDA (*P* < 0.05); 2 studies (Zhuang et al., 2017; Yang et al., 2018) reducing caspase-3 (*P* < 0.05); 4 studies (Hao et al., 2009, 2016; Xiong and Wei, 2015; Zhuang et al., 2017) increasing the ratio of Bcl-2/Bax protein (*P* < 0.05); 1 study (Hao et al., 2016) reducing expression level of p-Akt (*P* < 0.05); 1 study (Chai et al., 2018) decreasing content of Ca^2+^ in myocardium (*P* < 0.05); 2 studies (Bai et al., 2016; Zhang et al., 2018) decreasing expression of NF-κB; 1 study (Xiong and Wei, 2015) decreasing the level of p-ErK, p-JNK, p-p38MAPK; 1 study (Zhang et al., 2018) suppressing PMNs infiltration (*P* < 0.05); and 1 study (Zhang et al., 2018) decreasing TNF-α and IL-6 (*P* < 0.05).

## Discussion

### Summary of Evidence

In the present study, 19 studies with total 437 animals were included. GB exerted potential cardioprotective function, largely through antioxidant, anti-inflammation, anti-apoptosis and improvement of energy metabolism.

### Possible Mechanism

In the early phase of reperfusion, the ischemic myocardium generates oxidative stress. Oxidative stress can increases the release of oxidative free radicals production such as ROS, RNS, and OH and decreases the ability of free radicals scavenging, which further leads to cardiomyocyte cell injury (Navarro-Yepes et al., 2014). The excessive production of oxidative free radicals in the cellular and subcellular levels peroxidates the lipid, opens the mitochondrial permeability transition pore and dysfunctions the sarcoplasmic reticulum, resulting in overloaded intracellular Ca^2+^ and damaged cell membrane (Hausenloy and Yellon, 2013). Therefore, decreasing the accumulation of oxygen free radicals is an important approach to protect against myocardial I/R injury. The present study showed that GB could increase the level of antioxidant enzymes such as SOD, GSH-Px and CAT, maintain the dynamic balance of oxygen free radicals formation, restrain trigger of lipid peroxidation, protect biomembranes' integrity and function, and decrease the release of MDA from mitochondria, thereby exerts cardioprotective effect in through antioxidation.

Myocardial I/R damages the myocardium through inflammatory response (Marchant et al., 2012). During the processes of I/R injury, NF-κB pathway (Wang et al., 2007), which is critical to the inflammation and apoptosis during various diseases, mediates the upregulated expression of various pro-inflammatory cytokines such as TNF-α, IL-6, and IL-1β (Ma et al., 2014). In addition, the upstream factors of NF-kB—mitogen-activated protein kinase (MAPK) signaling pathway, including p38 MAPK, ERK, and JNK, also participates in regulating the inflammation (Chung et al., 2012; Himaya et al., 2012). The present study showed that GB exerted anti-inflammatory effect via significantly inhibiting the expression of NF-κB proteins, reducing the level of phosphorylation of p38, Jnk, and Erk protein, and decreasing the expression of TNF-a and IL-6 compared with control.

Myocardial I/R sequentially induce the cardiomyocyte Ca^2+^ overload, oxidative stress, opening of the mitochondrial permeability transition pore, mitochondrial membrane depolarization and uncoupling of oxidative phosphorylation, and ATP depletion and cell death (Hausenloy and Yellon, 2003; Murphy and Steenbergen, 2008). The present study found that GB could improve the activity of Na^+^-K^+^-ATPase and Ca^2+^-Mg^2+^-ATPase, decrease the content of Ca^2+^ in myocardium, protect mitochondrial respiratory activity, reduce the accumulation of free fatty acid, lactic acid, elevate the ratio of ATP/AMP, maintain the homeostasis, and improve amplitude of cardiomyocyte shortening by modulating cardiac Ca^2+^ regulatory proteins such as SERCA2a, phospholamban, and Na^+^-Ca^2+^exchanger.

**Figure 9:**
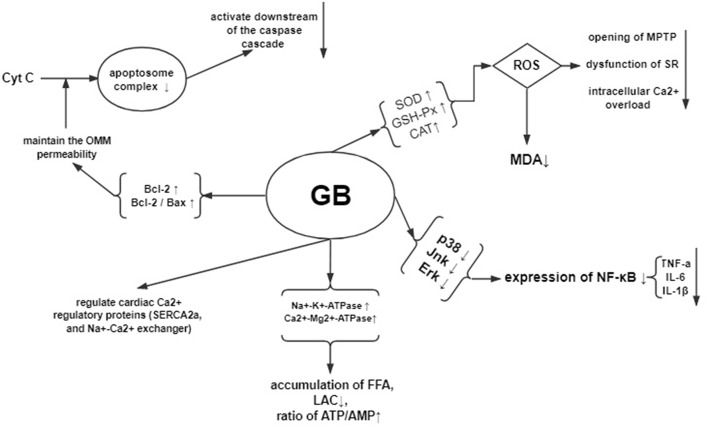
Mechanisms of GB exerted cardioprotective effects in myocardial ischemia/reperfusion injury.

Myocardial I/R injury resulted in cardiomyocyte apoptosis, which contributed to infarct size and ventricular dysfunction (Movassagh and Foo, 2008). The Bcl-2 family proteins were related to regulating the outer mitochondrial membrane (OMM) permeability. Increasing of OMM permeability caused by Bax could induce apoptosis, whereas decreasing the permeability caused by Bcl-2 could inhibit apoptosis (Ulgen et al., 2011). The present study indicated that GB could increase the expression of Bcl-2, decrease the expression of Bax, thus upregulate the ratio of Bcl-2/Bax to maintain the permeability of mitochondria, to inhibit activation of caspase-3, to inhibit the release of cytochrome C, to regulate intracellular free calcium concentration, and to reduce the activation and infiltration of macrophage, suggesting that GB may alleviate the myocardial I/R injury by anti-apoptosis. A summary of the cardioprotective mechanism of GB is provided in schematic (Figure 9).

### Limitations

Firstly, only Chinese and English studies were included, potentially causing certain bias (Guyatt et al., 2011). Secondly, the methodological quality score was generally moderate. Items identified as the core standards of research design such as allocation concealment, blinded assessment of outcome and the sample size calculation were failed to describe. The moderate methodological quality may affect the accuracy of the results (Landis et al., 2012; Macleod et al., 2015). Finally, 4 studies (Rioufol et al., 2003; Zhang et al., 2005; Zhao et al., 2014; Cao et al., 2016) used female animals. The response and sensitivity of experimental animals with the different gender to drugs and stimuli may be different, which could influence the final experimental results (Bae and Zhang, 2005).

### Implications

MI size is strongly associated with progressive worsening of left ventricular function and congestive heart failure, and enhance the long-term morbidity and mortality. Therefore, MI size can be used in clinical studies as a substitute endpoint for clinical outcomes and as an important prognostic measure when caring for patients with AMI (Ndrepepa et al., 2017). Physicians are striving to reduce the MI size so as to improve the prognosis of patients (Begley and Ioannidis, 2015). In animal studies, MI size is the robust primary outcome that invariably used to evaluate the efficacy of various pharmacological or non-pharmacological strategies that prevent I/R injury (Zhou et al., 2018). In the present study, the results showed that GB could reduce the MI size, improve the diastolic and systolic function, decrease the level of CK, AST, and LDH, increase the level of GSH-Px. Thus, our study is valuable to the translational development.

Animal studies are mainly targeted toward enriching our understanding of the mechanisms, etiology of human diseases, and forming the foundation on which future studies are built (Begley and Ioannidis, 2015). Accurate and complete reporting of animal studies is important to support the valid and reproducible research, while lower-quality reporting is related to deficiencies in experimental design that introduce bias and exaggerate effect sizes in the literature (Leung et al., 2018). The poor experimental design would restrain the translational development from animal studies into the clinical trials (Briel et al., 2013). The ARRIVE (Animals in Research: Reporting *In Vivo* Experiments) guidelines consist of a 20-item checklist published in 2010 with the aim of promoting the transparency and accuracy of animal research reporting, and of maximizing the availability and utility of information gained from every experiment (Gulin et al., 2015; Liu et al., 2016). In the present study, owing to the methodological quality, the results should be interpreted with caution. Thus, we proposed that further experiment design and reporting of the animal studies should refer to the ARRIVE guidelines (Kilkenny et al., 2010).

Inappropriate selection of the animal models could contribute to spurious or inconsistent results, as well as unnecessary animal use (Lecour et al., 2014). Given that animals with complications or risk factors such as ageing, diabetes and hypertension are close to the patients with myocardial infarction amalgamated with hypertension, hyperlipidemia, diabetes, insulin resistance, heart failure, altered coronary circulation and aging, we advise that further studies should use the appropriate animals instead of the young and healthy ones (Ferdinandy et al., 2014). These cardiovascular risks and comorbidities need to treat both short and long term, and contribute to the development of IR injury and complicate the therapy (Rossello and Yellon, 2016).

Animal models are often used to study the effectiveness of a drug or procedure and explore the mechanisms before proceeding to clinical trial (Roberts et al., 2002; Liao et al., 2012), which can be divided into *in vivo* and *ex vivo* models. The environment in which the *in vivo* myocardial infarction model located is exposed to normal humoral influences and neuronal regulation, and thus *in vivo* models can fully test the effect of the drug as an organic whole. However, many internal factors act as confounders, resulting in the deficiencies of the drug's effectiveness. In addition, the process of ligating the coronary artery and putting it back into the body needs high-tech requirements and high costs. In comparison, the isolated heart model with its broad spectrum of physiological, biochemical, and morphological measurements is a powerful *ex vivo* cardiovascular research tool that can be applied under numerous pathological condition (Skrzypiec-Spring et al., 2007; Liao et al., 2012). It possesses high-reproducibility, convenient operation, accurate results and time-saving due to the relatively simple process. Meanwhile, it is conducive to explore the mechanisms because various influencing factors can be strictly controlled easily. Compared with the cell models, the isolated heart model provides a more realistic correlation with the *in vivo* studies (Nahar et al., 2013). However, since the experimental environment of the *ex vivo* model can't fully simulate the internal environment, the results may not be comprehensive. According to the characteristics of animal models, *in vivo* experiments are mainly used to study the pharmacodynamics of pharmaceuticals, while the e*x vivo* experiments are more commonly contributed to explore mechanisms and physiological processes. This may explain the reason why the effect on infarct size *in vivo* heart model (Bai et al., 2016; Cao et al., 2016; Meng, 2017; Zhuang et al., 2017) showed better effects compared with the isolated heart model in the present study (Hao et al., 2009, 2016). Future experiments can select different models for different experimental purposes. However, no single experimental model is sufficient to investigate of the mechanism and therapeutic effects of the drug simultaneously (Jiang et al., 2016). In order to test the drug research systematically and deeply, we strongly suggest to utilize two models that complement each other.

The group with more times (≥5) of drug administration (Bai et al., 2016; Meng, 2017; Zhuang et al., 2017) showed a better outcome than that with fewer times (<5) (Hao et al., 2009, 2016; Cao et al., 2016). The pharmacological effect of TCM comes from biologically active part or active chemical components, which demonstrate the preventive and therapeutic effects. The bioactive ingredients of TCM are very complex and totally different from that of modern western medicine. The research on a scientific theory about TCM's pharmacological effects and bioactive ingredients is limited (Huang et al., 2015). The bioavailability of active ingredients in TCM is low, and their affinity is weak (Yu and Lu, 2018). The effect of drug treatment is relatively slow, which requires a certain period of treatment. GB is a pure compound of the Ginkgo biloba leaves. In the present study, 3 studies (Bai et al., 2016; Meng, 2017; Zhuang et al., 2017) were administrated GB, qd, for 7 days, whereas other 3 studies (Hao et al., 2009, 2016; Cao et al., 2016) were administrated GB only 1 time. The drug concentration in the body increases proportionally when the dosage of the drug is administrated more times. Repeated administration of drugs would help to achieve and maintain the effective blood concentrations. Therefore, further studies administer GB for multiple times.

GB was an antagonist of PAF that inhibits platelet activation/aggregation via competitive binding to PAF receptor (Heinroth-Hoffmann et al., 1990). Owing to the slow onset time of herbal efficacy, administration of herbs before the induction of models was often used for herbal pharmacological researches in order to reach the effective plasma concentration. The treatment post-model induction is more in line with clinical practice. Thus, it is more appropriate to study herbal pharmacology in both two different administrations. The results of present study showed that the efficacy in post-model induction group were better than that in the pre-model induction group, suggesting that the GB has fast onset time of decreasing platelet function directly via inhibiting the effect of PAF to alleviate myocardial I/R injury. Further study of this novel issue is needed.

Mechanisms underlying the pathogenesis of myocardial I/R injury are particularly complex, multifactorial and highly interconnected (Russo et al., 2017). The oxidative stress, apoptosis, energy metabolism disorder, and inflammation were described above. Other mechanisms such as Calcium paradox, pH paradox, metabolic modulation, Magnesium therapeutic, hypothermia, and autophagy, were also related to reperfusion injury. Based on the understanding of the pathophysiology of I/R injury, several advance non-pharmacological and pharmacological cardioprotective strategies were proposed to protect the ischemic myocardium in recent years. About non-pharmacological therapy, studies in animal models showed that ischemic pre-and post-conditioning, remote ischemic conditioning and hypothermia potentially stimulate the cardioprotective response. Meanwhile, different cardioprotective pharmacological agents have been confirmed in preclinical studies. These pharmacological agents include agonists of β-adrenoceptors (β-AR) or adenosine receptors, L-type voltage-dependent Ca^2+^ channels (VDCC) or mitochondrial Ca^2+^ uniporter (MCU) blockers, anti-platelet, modulators of NO biosynthesis or Na^+^/Ca^2+^ exchanger (NCX), resveratrol, methylene blue and intestinal lipase inhibitors (Caricati-Neto et al., 2019). In the future, nanotechnology-guided drug delivery systems will become new direction (Minamino, 2012), and novel cardioprotective mechanisms of GB should be investigated.

Assessing the risk of bias of the individual studies is a key feature of a systematic review. The Cochrane risk of bias (RoB) Tool is developed by the Cochrane Collaboration to establish consistency and avoid discrepancies in assessing the methodological quality of RCTs (Hooijmans et al., 2014). Analogously, when conducting systematic review of preclinical studies, the main difference is the methodological quality assessment methods. Some checklists have been developed to evaluate the quality of *in vivo* animal intervention experiments (Hooijmans et al., 2014; Zhang et al., 2017), including the SYstematic Review Centre for Laboratory animal Experimentation (SYRCLE)'s RoB tool and the Collaborative Approach to Meta-Analysis and Review of Animal Data from Experimental Studies (CAMARADES) 10-item checklist. About *in vitro* studies, the ROB assessment can refer to 10-item scale developed by our group (Bao et al., 2018). However, there are no quality assessment tools for a systematical review of *ex vivo* studies. The 9 out of 10 items of CAMARADES checklist are involved in the process of *ex vivo* studies except an item of animal model. Therefore, it is appropriate that we choose the CAMARADES 10-item list to assess the quality of *ex vivo* studies. The *ex vivo* studies are commonly contributed to explore mechanisms and physiological processes; however, many experimental did not choose animal models with comorbidities such as diabetes, hypertension, and atherosclerosis. When using the CAMARADES 10-item checklist to evaluate the quality of study, the point of appropriate animal model did not award that potentially caused bias accordingly. Thus, further *ex vivo* studies should choose appropriate models that make the results more accurate and comprehensive so as to improve efficiency in the design of study and promote the translation of findings from animal studies into clinic.

## Conclusion

The present study demonstrated that GB could alleviate the myocardial I/R injury, mainly through the antioxidation, anti-inflammation, anti-apoptosis, and the improvement of energy metabolism.

## Data Availability Statement

The raw data supporting the conclusions of this manuscript will be made available by the authors, without undue reservation, to any qualified researcher.

## Author Contributions

YW, GZ, and P-CZ contributed to the study conception and design. P-CZ, QT, ZZ, Z-HW, and L-HD contributed to the acquisition, the analysis, and/or the interpretation of data. GZ and YW gave the final approval and contributed overall responsibility for this published work.

### Conflict of Interest

The authors declare that the research was conducted in the absence of any commercial or financial relationships that could be construed as a potential conflict of interest.
